# An Investigation of N-Hydroxyphthalimide Catalyzed Aerobic Oxidation of Toluene without Metal Ions in Liquid Phase: Effect of Solvents and Phase Transfer Catalysts

**DOI:** 10.3390/molecules29133066

**Published:** 2024-06-27

**Authors:** Guojun Shi, Longsheng Dong, Ya Feng

**Affiliations:** School of Chemistry and Chemical Engineering, Yangzhou University, Yangzhou 225002, China; mx120230476@stu.yzu.edu.cn (L.D.); yafeng0327@163.com (Y.F.)

**Keywords:** toluene, oxidation, N-hydroxyphthalimide, solvent, phase transfer catalyst

## Abstract

The selective oxidation of toluene to yield value-added oxygenates, such as benzyl alcohol, benzaldehyde, and benzoic acid, via dioxygen presents a chlorine-free approach under benign conditions. Metal-free catalytic processes are preferred to avoid metal ion contamination. In this study, we employed N-hydroxyphthalimide (NHPI) as a catalyst for the aerobic oxidation of toluene to its oxygenated derivatives. The choice of solvent exerted a significant impact on the catalytic activity and selectivity of the catalyst NHPI at reaction temperatures exceeding 70 °C. Notably, hexafluoroisopropanol substantially enhanced the selective production of benzaldehyde. Furthermore, we identified didecyl dimethyl ammonium bromide, featuring two symmetrical long hydrophobic chains, as a potent enhancer of NHPI for the solvent-free aerobic oxidation of toluene. This effect is ascribed to its unique symmetrical structure, extraction capabilities, and resistance to thermal and acid/base conditions. Based on the product distribution and control experiments, we proposed a plausible reaction mechanism. These findings may inform the industrial synthesis of oxygenated derivatives from toluene.

## 1. Introduction

The selective oxidation of toluene yields valuable organic compounds such as benzyl alcohol, benzaldehyde, and benzoic acid [1,2]. Particularly, direct oxofunctionalization of toluene’s primary C−H bonds streamlines the synthetic pathway [3] and offers a template for converting a range of compounds with benzylic C−H bonds into their oxygenated counterparts [4]. Industrially, toluene chlorination followed by subsequent hydrolysis is commonly employed to produce benzyl alcohol, benzaldehyde, and benzoic acid [5]. Nonetheless, this method often leads to substantial equipment corrosion [6,7], driving both industry and academia to develop a selective oxidation process under mild conditions.

Molecular oxygen, as an oxidant, is cost-effective, environmentally benign, and readily available [8,9]. However, its low reactivity with alkyl-substituted aromatics, due to spin-flip restrictions between triplet molecular oxygen and sp^3^ hybridized C−H bonds, is challenging [10,11]. Elevating the reaction temperature accelerates substrate oxidation but compromises selectivity for the target organic compounds [12,13]. A carefully designed catalyst can facilitate the efficient conversion of toluene to its value-added derivatives under gentle conditions.

Vapor-phase oxidation is preferred for toluene oxidation because it allows for continuous operation and high space–time yields. Yet, to minimize overoxidation to useless byproducts, single-pass conversion must be limited [14]. Liquid phase oxidation, conducted at lower temperatures, reduces unwanted overoxidation [15]. Numerous studies have aimed to enhance the conversion of toluene to oxygenated derivatives [16]. However, issues such as low catalytic activity, reactor corrosion, product contamination with metal ions, and challenges in catalyst recovery persist. Consequently, many researchers have concentrated on the solvent-free selective aerobic oxidation of toluene, recognizing it as an efficient and benign approach [17,18].

N-hydroxyphthalimide (NHPI) has proven to be highly efficient and versatile in catalyzing the transformation of alkyl aromatics into value-added compounds through in situ-generated phthalimide N-oxyl (PINO) radicals in the presence of an initiator [19]. Our findings indicate that NHPI/Co(II) combination effectively promotes the conversion of toluene, ethylbenzene, and cumene to benzaldehyde, acetophenone, and cumyl hydroperoxide, respectively, using dioxygen at room temperature [16,20,21]. However, the use of metal ions as initiators poses challenges in separation, recovery, and potential product contamination. Consequently, we hypothesize that NHPI could catalyze the aerobic oxidation of alkyl aromatics without an initiator through a single electron transfer (SET) between molecular oxygen and NHPI at elevated temperatures, leading to the formation of active PINO radicals [22].

In this study, we employ NHPI to independently catalyze the aerobic oxidation of toluene to its oxygen-containing derivatives. We investigate different solvents to elucidate their effects on the oxidation process and introduce phase transfer catalysts to facilitate the transformation under solvent-free conditions. Moreover, we construct the reaction network for toluene oxidation through control experiments and propose plausible pathways to elucidate the observed catalytic outcomes.

## 2. Results and Discussion

### 2.1. Effect of Solvents on Metal-Free Oxofunctionalization of Toluene

**Figure 1:**
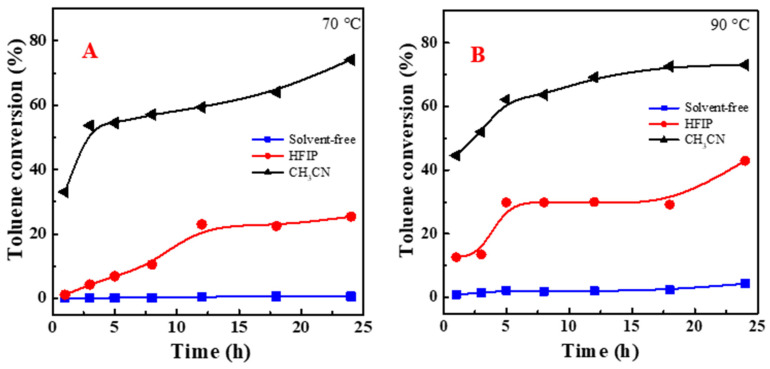
Effect of solvents on toluene conversion at 70 °C (**A**) and 90 °C (**B**). Toluene (2 mmol); NHPI (0.05 mmol); solvent (40 mmol). HFIP: hexafluoroisopropanol, CH_3_CN: acetonitrile.

**Figure 2:**
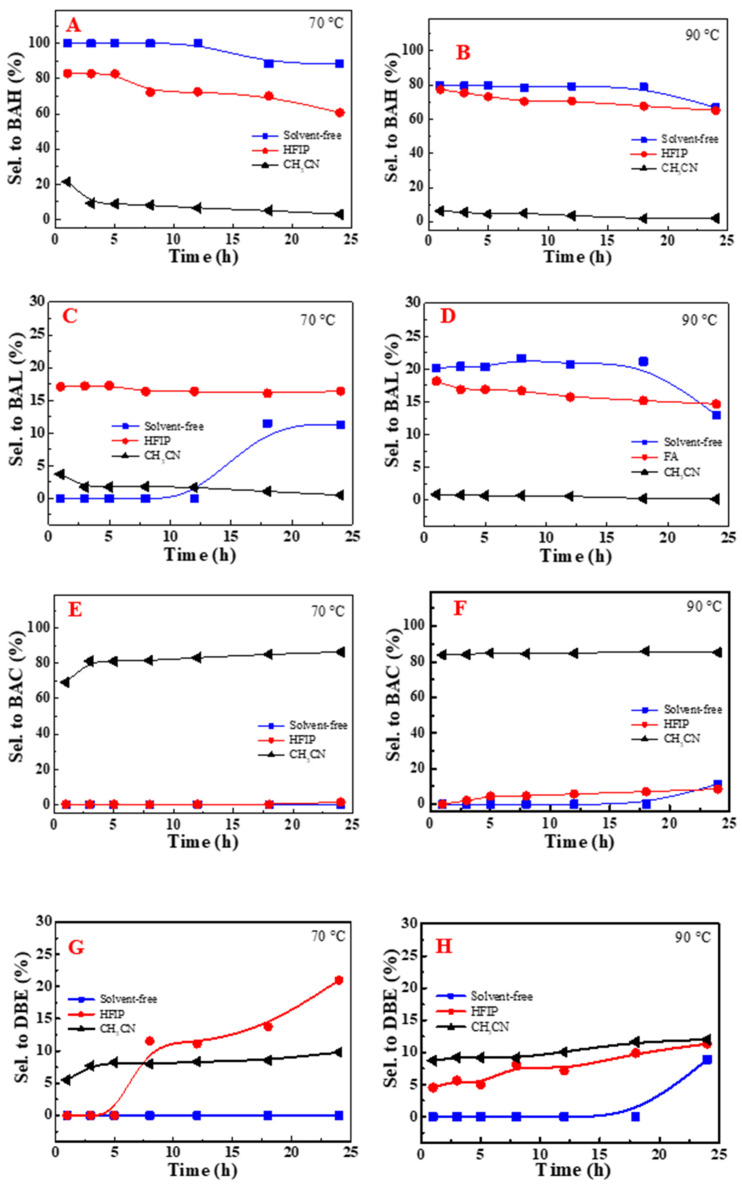
Effect of solvent on selectivities to benzaldehyde (**A**,**B**), benzyl alcohol (**C**,**D**), benzoic acid (**E**,**F**), and dibenzyl ether (**G**,**H**) in CH_3_CN and HFIP and under solvent-free conditions. HFIP: hexafluoroisopropanol; CH_3_CN: acetonitrile; benzaldehyde: BAH; BAL: benzyl alcohol; BAC: benzoic acid; DBE: dibenzyl ether; Sel.: selectivity.

**Figure 3:**
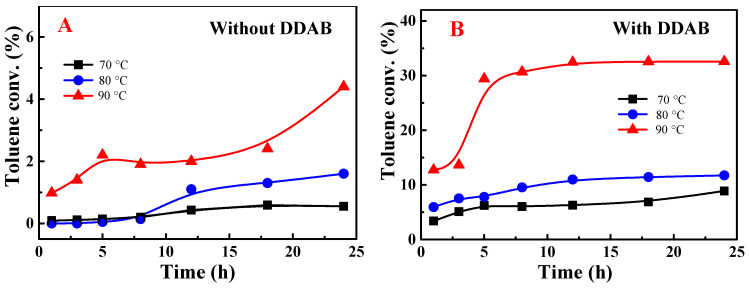
Promotional role of the phase transfer catalyst DDAB of NHPI-catalyzed toluene oxidation under solvent-free conditions (without (**A**) and with (**B**) DDAB). Conv.: conversion; NHPI: N-hydroxyphthalimide; DDºAB: didecyl dimethyl ammonium bromide.

The aerobic oxidation of toluene was conducted using various solvents, with results analyzed through GC-MS, as depicted in Figure 1, Figure 2 and Figure 3. Figure 1 illustrates the impact of solvents on the aerobic oxidation process. In the absence of solvents, toluene exhibited negligible reactivity, likely due to the poor solubility of the catalyst NHPI, as shown in Figure 1A. A marked enhancement in toluene conversion was noted with the introduction of hexafluoroisopropanol (HFIP) as a solvent. Specifically, after a 12 h reaction period, the conversion of toluene exceeded 20%. This improvement might be attributed to the solvents’ promotional effects on both the solubility and the catalytic transformation of toluene, as referenced in studies [23,24]. Utilizing acetonitrile as the solvent resulted in a reduced induction period and increased reactivity, paralleling observations from previous research on cumene oxidation, which could be due to the solvent’s high polarity [25]. Toluene conversions were increased in acetonitrile and hexafluoroisopropanol at an elevated reaction temperature (Figure 1B). Compared with acetonitrile, hexafluoroisopropanol exhibited stronger promotion on toluene oxidation. More than 40% of toluene was converted, and the predominant product was benzaldehyde (Figure S4). There was a negligible increase in toluene conversion under solvent-free conditions when the reaction temperature was increased from 70 °C to 90 °C.

Figure 2 illustrates the selectivity variations for benzaldehyde, benzyl alcohol, benzoic acid, and dibenzyl ether when employing different solvents. Observations indicate that benzoic acid and dibenzyl ether are the primary products in acetonitrile, with their selectivities showing an upward trend as the reaction time is prolonged. Utilizing hexafluoroisopropanol (HFIP) as the solvent, the initial catalytic reaction phase exhibited a selectivity for benzaldehyde exceeding 80%. This elevated selectivity is attributable to the formation of a hydrogen-bonded adduct between the target benzaldehyde and HFIP, which inhibits the further oxidation of benzaldehyde to benzoic acid. Nonetheless, the diminishing benzaldehyde selectivity in HFIP over time might be due to a concurrent rise in dibenzyl ether selectivity. The differences in selectivities are not of significance under solvent-free conditions due to a very low toluene reactivity. More catalytic tests were performed to justify whether temperature is more important than NHPI, and the results are in Table S1. It is noticed that there was poor reactivity of toluene observed in the absence of the catalyst NHPI even at 90 °C, indicating that the toluene oxidation observed can be ascribed to the catalytic role of NHPI.

Control experiments utilizing various starting materials were conducted to elucidate the reaction mechanisms in HFIP, with the findings presented in Table 1. It was observed that benzaldehyde underwent transformation into benzyl alcohol, benzoic acid, and dibenzyl ether, achieving a 63.3% conversion rate. The formation of benzyl alcohol is likely attributable to the Cannizzaro reaction involving benzaldehyde. Benzoic acid may result from both the direct oxidation of benzaldehyde and its disproportionation, with the former being the predominant pathway. The genesis of dibenzyl ether is presumably through the reaction of benzyl alcohol. Surprisingly, benzaldehyde exhibited considerably higher reactivity in HFIP compared to the marked selectivity for this compound observed when toluene served as the starting material, as depicted in Figure 2. This indicates that HFIP’s role as a solvent is not the sole factor influencing the selective generation of benzaldehyde. Benzyl alcohol displayed lower reactivity, corresponding with the consistent selectivity toward the final product illustrated in Figure 2. Under the experimental conditions employed, benzoic acid was inert. Dibenzyl ether underwent transformation into benzaldehyde, benzyl alcohol, and benzoic acid, processes that can be attributed to its hydrolysis followed by further oxidation.

### 2.2. Role of Phase Transfer Catalysts on Metal-Free Oxofunctionalization of Toluene under Solvent-Free Conditions

Solvents facilitate the dissolution of reactants and catalysts, thereby enhancing the efficiency of conversion. Nevertheless, their use is often marred by challenges in recovery and recycling, alongside potential losses and equipment erosion, incurring additional expenses. The selective aerobic oxidation of toluene into value-added organics is more advantageous under solvent-free conditions, as indicated in reference [26]. Figure 1 illustrates that NHPI exhibits minimal catalytic activity in the absence of a solvent, attributable to its poor solubility in the reactant toluene. To augment toluene conversion without solvents, phase transfer catalysts have been employed, with the outcomes examined via GC-MS (Figure S5) and delineated in Table 2.

The introduction of nonionic (OP-10) and anionic (SDBS) surfactants did not yield noticeable enhancements in solvent-free toluene oxidation, with conversions similar to those without surfactants (Entries 1−3). However, the incorporation of the zwitterionic surfactant (dodecyldimethylammonio)acetate (BS-2) significantly improved toluene conversion from 2.2% to 11.2%. This enhancement is likely due to BS-12′s facilitative effect on the dissolution of the catalyst NHPI. Notably, didecyl dimethyl ammonium bromide (DDAB), a cationic surfactant possessing two symmetric long hydrophobic chains, achieved a remarkable toluene conversion rate of 29.4%. This high conversion rate can be attributed to its unique symmetrical structure, extraction capabilities, and superior resistance to heat, acids, and bases during the liquid phase reaction [27,28]. In order to investigate the toluene derivates, the selective aerobic oxidation of paraxylene was performed and the results are shown in Table S2. It was found that there was relatively high conversion, and the predominant product is p-methyl benzoic acid. In the surfactant-mediated catalytic reaction, the primary products were benzaldehyde, benzyl alcohol, and benzoic acid, with no evidence of complete oxidation products, demonstrating an exceptional selectivity for oxidation.

Figure 3 depicts the influence of reaction temperature and time on the catalytic oxidation process in a solvent-free environment. In the absence of DDAB, toluene demonstrated a notably sluggish reaction rate, which persisted despite exposure to temperatures as high as 90 °C for durations up to 24 h. Introducing DDAB significantly enhanced the conversion of toluene, particularly within the temperature range of 70 °C to 90 °C. Remarkably, elevating the temperature to 90 °C markedly improved toluene’s reactivity, as evidenced by an S-shaped conversion time profile that typifies classic auto-oxidation kinetics.

**Figure 4:**
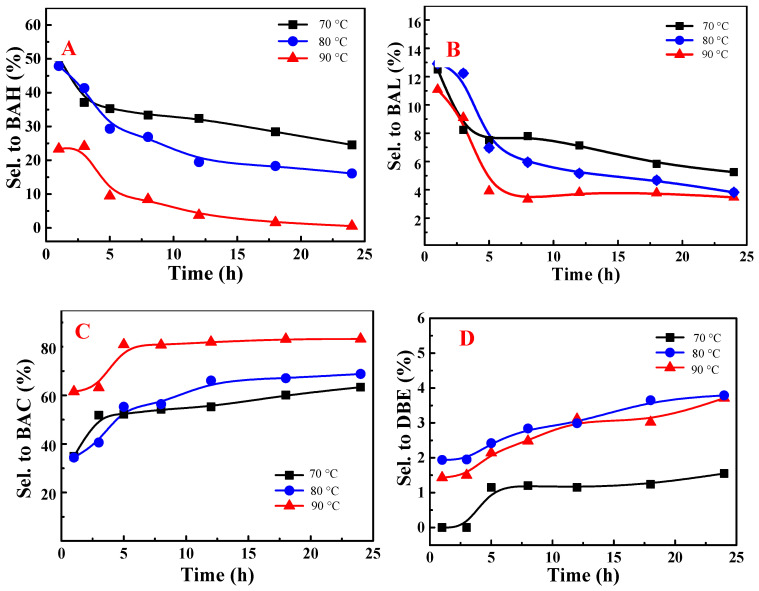
Effect of reaction temperature on selectivities to benzaldehyde (**A**), benzyl alcohol (**B**), benzoic acid (**C**), and dibenzyl ether (**D**) for NHPI/DDAB-catalyzed toluene oxidation under solvent-free conditions. NHPI: N-hydroxyphthalimide; DDAB: didecyl dimethyl ammonium bromide; BAH: benzaldehyde; BAL: benzyl alcohol; BAC: benzoic acid; DBE: dibenzyl ether; Sel.: selectivity.

Figure 4 illustrates the effects of reaction temperature and time on the selectivities during the NHPI-catalyzed aerobic oxidation of toluene. Initially, high selectivities for benzaldehyde and benzyl alcohol were observed, indicating that these products might be formed directly from toluene. An increase in both reaction temperature and duration resulted in reduced selectivities for benzaldehyde and benzyl alcohol and an enhanced selectivity for benzoic acid, likely due to their further oxidation to benzoic acid. Moreover, a rise in the selectivity for dibenzyl ether was noted with an elevated reaction temperature or extended time, potentially associated with the oxidation of benzyl alcohol.

Control experiments were conducted to elucidate the reaction pathway using benzyl alcohol and dibenzyl ether as substrates, as summarized in Table 3. Under the tested conditions, benzyl alcohol readily oxidizes to benzaldehyde, which further converts to benzoic acid. The formation of dibenzyl ether likely results from the etherification of benzyl alcohol, and its subsequent esterification with benzoic acid may yield benzyl benzoate. Additionally, dibenzyl ether demonstrated significant reactivity, achieving up to 96.9% conversion. This reactivity yielded benzaldehyde through direct oxidation, benzyl alcohol via hydrolysis, benzoic acid through indirect oxidation, and benzyl benzoate from esterification. However, the use of benzaldehyde and benzoic acid as initial reactants to assess their reactivity under solvent-free conditions was precluded due to safety and/or technical considerations.

**Figure 5:**
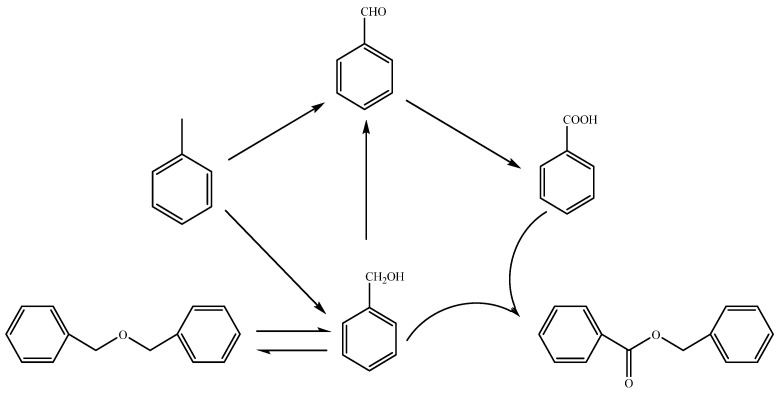
Reaction network of toluene oxidation catalyzed by NHPI under solvent-free conditions.

Based on the product distribution data presented in Table 2 and findings from the control experiments detailed in Table 3, we have deduced the reaction pathway for the solvent-free oxidation of toluene, catalyzed by NHPI and DDAB, as depicted in Figure 5. Toluene is initially oxidized to yield benzyl alcohol and benzaldehyde, with the latter potentially undergoing further oxidation to form benzoic acid. An esterification reaction between benzyl alcohol and benzoic acid may lead to the formation of benzyl benzoate. Additionally, benzyl alcohol can reversibly convert to dibenzyl ether.

Additional control experiments were conducted to elucidate the contribution of the catalyst combination, with the findings presented in Table 4. In the absence of any catalyst, toluene conversion was negligible. Individually, NHPI and DDAB demonstrated limited activity in catalyzing the oxidation of toluene. Notably, the synergy between NHPI and DDAB resulted in superior catalytic efficacy for the solvent-free oxidation of toluene using molecular oxygen, underscoring the remarkable enhancing effect of DDAB on the catalytic behavior of the polar NHPI catalyst. Furthermore, the introduction of a radical scavenger terminated the oxidation process, implying that the conversion of toluene proceeded through a free radical mechanism under the specified reaction conditions.

**Figure 6:**
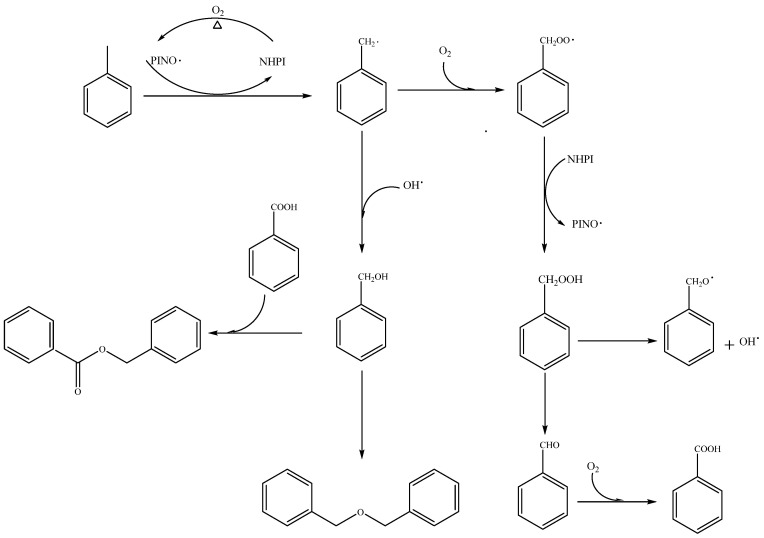
A possible reaction mechanism of toluene oxidation catalyzed by NHPI/DDAB under solvent-free conditions.

Based on the product distribution depicted in Figure 4 and data from control experiments presented in Table 3 and Table 4, Figure 6 illustrates a plausible reaction mechanism. N-hydroxyphthalimide (NHPI) is likely activated through a single electron transfer (SET) with dioxygen, forming the phthalimide N-oxy (PINO) radical. This radical initiates a chain of auto-oxidation reactions. The PINO radical abstracts a hydrogen atom from the substrate toluene, generating a benzyl free radical. This intermediate readily reacts with molecular oxygen, producing benzyl peroxide radicals. A subsequent hydrogen atom transfer from the benzyl peroxide radical to the catalyst (NHPI) leads to the formation of benzyl hydroperoxide, concurrently reactivating PINO. Studies suggest that the benzyl peroxide radical favors hydrogen abstraction from NHPI over toluene, elucidating the catalyst’s role in enhancing toluene oxidation [29,30]. Benzyl hydroperoxide undergoes dehydration to yield the target product, benzaldehyde, which may further oxidize to benzoic acid. Alternatively, benzyl hydroperoxide can decompose into benzyl oxyl and hydroxyl radicals. These radicals can either abstract hydrogen atoms from toluene or NHPI or combine with benzyl radicals, respectively, resulting in the production of benzyl alcohol. Benzyl alcohol may then be oxidized to benzaldehyde and benzoic acid or react with benzoic acid to form benzyl benzoate. Additionally, dibenzyl ether can form through the intermolecular dehydration of benzyl alcohol.

## 3. Experimental Section

### 3.1. Aerobic Oxidation of Toluene in Different Solvents

The metal-free aerobic oxidation of toluene was conducted in a 50 mL PTFE-lined autoclave to assess the impact of various solvents on the efficacy of the NHPI catalyst. For a standard test, toluene (2 mmol), NHPI (0.05 mmol), and solvents (40 mmol) were placed into the autoclave, which was subsequently purged with O_2_ (99.999%) to eliminate non-oxygen gases. The assessments were typically carried out at 90 °C for 5 h, with an initial O_2_ pressure of 2.6 MPa, under vigorous stirring at 700 rpm. The reaction mixtures were analyzed using gas chromatography (GC), featuring a capillary column (SGE ACR-10, 30 m × 0.22 mm, with a stationary phase thickness of 0.25 μm) and a flame ionization detector (FID). Manual injections of the samples (1 μL) into the GC were performed under a nitrogen flow rate of 100 mL/min with a split ratio of 100:1. The GC column began at an initial temperature of 160 °C for 2 min, followed by a temperature increase to 200 °C at a rate of 20 °C/min, and a final hold time of 6 min. All the catalytic experiments were replicated at least three times to ensure reliability.

### 3.2. Solvent-Free Aerobic Oxidation of Toluene Mediated by Different Phase Transfer Catalysts

The effects of phase transfer catalysts on the aerobic oxidation of toluene were assessed using a method similar to that described in Section 2.1. These evaluations proceeded under solvent-free conditions, employing 40 mmol of the substrate toluene, 4 mmol of the catalyst NHPI, and 0.2 mmol of phase transfer catalysts. An analogous analytical approach was adopted with the exception of diluting samples with acetone at a 1:1 ratio prior to injection.

### 3.3. Identification of Products and Intermediates by GC-MS

The qualitative analysis of the products and intermediates resulting from toluene oxidation was performed using a GC-MS system (Trace ISQ, ThermoFisher, Waltham, MA, USA) equipped with a DB-5 capillary column (30 m in length, 0.25 mm i.d., and 0.25 μm film thickness). Prior to the analysis, the samples obtained from the catalytic reactions were first filtered through a 0.22 μm mesh sieve. Subsequently, 1 μL of the sample was automatically injected into the GC using helium as the carrier gas at a flow rate of 100 mL/min and a split ratio of 100:1. The column temperature was initially set to 70 °C for 20 min, then ramped up to 200 °C at a rate of 20 °C/min, and held for an additional 3 min. The NIST database facilitated the identification of the extracted products and intermediates.

## 4. Conclusions

In this study, the aerobic oxidation of toluene to produce valuable oxygenated derivatives was explored using metal-free catalysts. NHPI was found to be active for this catalytic transformation at a reaction temperature above 70 °C, and solvents exhibited a predominant role on catalytic activity and selectivity. Hexafluoroisopropanol (HFIP) was found to significantly enhance the selective conversion of toluene to benzaldehyde. Moreover, the NHPI-catalyzed aerobic oxidation of toluene proceeded under solvent-free conditions when paired with phase transfer catalysts. The cationic surfactant didecyl dimethyl ammonium bromide (DDAB), featuring two symmetric long hydrophobic chains, markedly improved NHPI’s catalytic efficiency for the solvent-free aerobic oxidation of toluene. This effect is likely attributable to its unique symmetric structure, extraction capabilities, and resistance to thermal and acid/base conditions. A tentative reaction mechanism has been proposed on the basis of the observed product distribution and outcomes from control experiments.

## Figures and Tables

**Table 1:** Controlled experiments performed using different starting materials in HFIP.

Starting Material	Conversion (%)	Selectivity (%)				
		BAH	BAL	BAC	DBE	Others
BAH	63.3	-	1.7	94.6	3.5	0.2
BAL	6.0	100	-	0.0	0.0	0.0
BAC	0.0	-	-	-	-	-
DBE	33.2	71.8	23.6	4.0	-	0.6
Toluene	29.8	73.3	16.9	4.6	5.0	0.2

Reaction conditions: starting material (2 mmol), HFIP (40 mmol), NHPI (0.2 mmol), O_2_ (2 MPa), 90 °C, 5 h. NHPI: N-hydroxyphthalimide; HFIP: hexafluoroisopropanol; BAH: benzaldehyde; BAL: benzyl alcohol; BAC: benzoic acid; DBE: dibenzyl ether.

**Table 2:** Effect of different types of phase transfer catalysts on the performance of homogeneous NHPI for solvent-free oxidation of toluene.

Entry	Phase Transfer Catalyst	Toluene Conversion (%)	Selectivity (%)				
			BAH	BAL	BAC	DBE	Others
1	-	2.2	79.7	20.3	0.0	0.0	0.0
2	OP-10	2.3	80.1	19.9	0.0	0.0	0.0
3	SDBS	2.4	79.3	19.9	0.0	0.0	0.8
4	BS-12	11.2	26.9	6.1	59.6	5.1	2.4
5	DDAB	29.4	9.4	3.9	80.9	2.1	3.6

Reaction conditions: toluene (40 mmol), NHPI (4 mmol), DDAB (0.2 mmol), O_2_ (2 MPa), 90 °C, 5 h. NHPI: N-hydroxyphthalimide; BAH: benzaldehyde; BAL: benzyl alcohol; BAC: benzoic acid; DBE: dibenzyl ether; OP-10: octyl phenol polyoxyethylene ether-10; SDBS: dodecyl benzenesulfonic acid; BS-12: (dodecyldimethylammonio)acetate; DDAB: didecyl dimethyl ammonium bromide.

**Table 3:** Controlled experiment performed using different starting materials under solvent-free conditions.

Starting Material	Conversion (%)	Selectivity (%)					
		BAH	BAL	BAC	DBE	BBE	Others
Toluene	29.4	9.4	3.9	80.9	2.1	0.0	3.6
BAL	100	11.3	-	71.4	0.9	7.4	9.0
DBE	96.9	6.5	3.0	28.2	-	28.8	33.5

Reaction conditions: Starting material (40 mmol), NHPI (4 mmol), DDAB (0.2 mmol), O_2_ (2 MPa), 90 °C, 5 h. NHPI: N-hydroxyphthalimide; DDAB: didecyl dimethyl ammonium bromide; BAH: benzaldehyde; BAL: benzyl alcohol; BAC: benzoic acid; DBE: dibenzyl ether; BBE: benzyl benzoate.

**Table 4:** Controlled experiments performed using different catalysts under solvent-free conditions ^a^.

Catalyst	Conversion (%)	Selectivity (%)				
		BAH	BAL	BAC	DBE	Others
None	0.3	86.8	13.2	0.0	0.0	0.0
NHPI	2.2	79.7	20.3	0.0	0.0	0.0
DDAB	0.2	100	0.0	0.0	0.0	0.0
NHPI, DDAB	29.4	9.4	3.9	80.9	2.1	3.6
NHPI, DDAB, TEMPO ^b^	0.0	-	-	-	-	-

^a^ Reaction conditions: toluene (40 mmol), NHPI (4 mmol), DDAB (0.2 mmol), O_2_ (2 MPa), 90 °C, 5 h. ^b^ TEMPO (40 mmol). TEMPO: 2,2,6,6-tetramethylpiperidinooxy; NHPI: N-hydroxyphthalimide; DDAB: didecyl dimethyl ammonium bromide; BAH: benzaldehyde; BAL: benzyl alcohol; BAC: Benzoic acid; DBE: dibenzyl ether.

## Data Availability

Data will be made available on request.

## Supplementary Materials

The following supporting information can be downloaded at: https://www.mdpi.com/article/10.3390/molecules29133066/s1. Figure S1: Chromatographic elution curve of the reaction solution in HFIP via GC-MS. Reaction conditions: toluene (2 mmol), HFIP (40 mmol), NHPI (0.2 mmol), O2 (2 MPa), 90 °C, 5 h. 1: benzaldehyde; 2: phenol; 3: benzyl alcohol; 4: o-benzyl hydroxylamine; 5: phenylmethoxyure; 6: o-phthalic anhydride; 7: phthalimide; 8: 1-benzyl-3-methylbenzene; 9: benzyl benzoate; 10: phenethoxybenzene; 11: N-hydroxyphthalimide. Figure S2: Chromatographic elution curve of the reaction solution of toluene in CH3CN via GC-MS. Reaction conditions: toluene (2 mmol), CH3CN (40 mmol), NHPI (0.2 mmol), O2 (2 MPa), 90 °C, 5 h. CH3CN: acetonitrile; NHPI: N-hydroxyphthalimide; 1: benzaldehyde; 2: benzyl alcohol; 3: benzyl formate; 4: 2-nitrobenzyl alcohol; 5: benzoic acid; 6: o-phthalic anhydride; 7: 2-phenylmethoxycarbonylbenzoic acid; 8: phthalimide; 9: N-hydroxyphthalimide; 10: o-tolyl benzoate; 11: 2-(4-methylphenyl)isoindole-1,3-dione; 12: 2-[(3-methylphenyl)carbamoyl]benzoic acid; 13: N-benzylphthalimide. Figure S3: Chromatographic elution curve of the reaction solution of toluene under solvent-free conditions via GC-MS. Reaction conditions: toluene (40 mmol), NHPI (4 mmol), O2 (2 MPa), 90 °C, 5 h. 1: toluene; 2: chlorobenzene; 3: benzaldehyde; 4: benzyl alcohol. Figure S4: Effect of reaction time on toluene conversion (A) and the selectivity to benzaldehyde (B) in HFIP. Conv.: conversion; Sel.: selectivity; BAH: benzaldehyde. Table S1. Catalyst controlled experiment in HFIP. Figure S5: Chromatographic elution curve of the reaction solution of toluene oxidation catalyzed by NHPI/DDAB under solvent-free conditions via GC-MS. Reaction conditions: toluene (40 mmol), NHPI (4 mmol), DDAB (0.2 mmol), O2 (2 MPa), 90 °C, 5 h. NHPI: N-hydroxyphthalimide; DDAB: didecyl dimethyl ammonium bromide; 1: benzaldehyde; 2: benzonitrile; 3: benzyl alcohol; 4: methyl formate; 5: 2-nitrobenzyl alcohol; 6: benzoic acid; 7: o-phthalic anhydride; 8: phthalimide; 9: N-hydroxyphthalimide; 10: (4-methylphenyl)benzoate; 11: benzyl benzoate; 12: o-tolyl benzoate. Table S2: The conversion and selectivities for selective aerobic oxidation of paraxylene catalyzed by the binary NHPI/DDAB under solvent-free conditions.

## References

[1] Wang X.L., Wu G.D., Wang F., Liu H., Jin T.F. (2017). Solvent-free selective oxidation of toluene with O_2_ catalysed by anion modified mesoporous mixed oxides with high thermal stability. Catal. Commun..

[2] Xu J., Shi G., Liang Y., Lu Q., Ji L. (2021). Selective aerobic oxidation of toluene to benzaldehyde catalyzed by covalently anchored N-hydroxyphthalimide and cobaltous ions. Mol. Catal..

[3] Deng C., Cui Y., Chen J., Chen T., Guo X., Ji W., Peng L., Ding W. (2021). Enzyme-like mechanism of selective toluene oxidation to benzaldehyde over organophosphoric acid-bonded nano-oxide. Chin. J. Catal..

[4] Che C.M., Lo V.K.Y., Zhou C.Y., Huang J.S. (2011). Selective functionalisation of saturated C–H bonds with metalloporphyrin catalysts. Chem. Soc. Rev..

[5] Gast S., Tuttlies U.S., Nieken U. (2020). Kinetic study of the toluene oxidation in homogeneous liquid phase. Chem. Eng. Sci..

[6] Mal D.D., Khilari S., Pradhan D. (2018). Efficient and selective oxidation of toluene to benzaldehyde on manganese tungstate nanobars: A noble metal-free approach. Green Chem..

[7] Martins N.M.R., Pombeiro A.J.L., Martins L.M.D.R.S. (2018). A green methodology for the selective catalytic oxidation of styrene by magnetic metal-transition ferrite nanoparticles. Catal. Commun..

[8] Mu C., Cao Y., Wang H., Yu H., Peng F. (2018). A kinetics study on cumene oxidation catalyzed by carbon nanotubes: Effect of N-doping. Chem. Eng. Sci..

[9] Jiang J., Luo R., Zhou X., Wang F., Ji H. (2017). Metalloporphyrin-mediated aerobic oxidation of hydrocarbons in cumene: Co-substrate specificity and mechanistic consideration. Mol. Catal..

[10] Miao C., Zhao H., Zhao Q., Xia C., Sun W. (2016). NHPI and ferric nitrate: A mild and selective system for aerobic oxidation of benzylic methylenes. Catal. Sci. Technol..

[11] Huang H., Ye W., Song C., Liu Y., Zhang X., Shan Y., Ge Y., Zhang S., Lu R. (2021). Confinement of Au^3+^-rich clusters by using silicalite-1 for selective solvent-free oxidation of toluene. J. Mater. Chem. A.

[12] Yoshino Y., Hayashi Y., Iwahama T., Sakaguchi S., Ishii Y. (1997). Catalytic oxidation of alkylbenzenes with molecular oxygen under normal pressure and temperature by N-hydroxyphthalimide combined with Co(OAc)_2_. J. Org. Chem..

[13] Opeida I.A., Plekhov A.L., Kushch O.V., Kompanets M.A. (2012). On the mechanism of oxidation process initiation by the N-hydroxyphthalimide-cobalt (II) acetate system. Russ. J. Phys. Chem. A.

[14] Bertolini G.R., Pizzio L.R., Kubacka A., Batista M.J.M., Garcia M.F. (2018). Composite H_3_PW_12_O_40_-TiO_2_ catalysts for toluene selective photo-oxidation. Appl. Catal. B.

[15] Ishii Y., Sakaguchi S., Iwahama T. (2010). Innovation of hydrocarbon oxidation with molecular oxygen and related reactions. Adv. Synth. Catal..

[16] Shi G., Lu Q., Xu J., Wang J., Ji J. (2021). Co-immobilization of N-hydroxyphthalimide and cobaltous ions as a recyclable catalyst for selective aerobic oxidation of toluene to benzaldehyde. J. Environ. Chem. Eng..

[17] Deng W., Wan Y.P., Jiang H., Luo W.P., Tan Z., Jiang Q., Guo C.C. (2014). Solvent-free aerobic oxidation of toluene over metalloporphyrin/NHPI/CTAB: Synergy and mechanism. Catal. Lett..

[18] Yang H.M., Wu H.S. (2003). Interfacial mechanism and kinetics of phase-transfer catalysis. Catal. Rev..

[19] Kurganova E.A., Sapunov V.N., Koshel G.N., Frolov A.S. (2016). Selective aerobic oxidation of cyclohexyl- and sec-alkylarenes to hydroperoxides in the presence of N-hydroxyphthalimide. Russ. Chem. Bull..

[20] Shi G., Feng Y., Xu S., Lu Q., Liang Y., Yuan E., Ji L. (2021). Covalent anchoring of N-hydroxyphthalimide on silica via robust imide bonds as a reusable catalyst for the selective aerobic oxidation of ethylbenzene to acetophenone. New J. Chem..

[21] Lu Q., Shi G., Zhou H., Yuan E., Chen C., Ji L. (2022). A highly efficient transformation from cumene to cumyl hydroperoxide via catalytic aerobic oxidation at room temperature and investigations into solvent effects, reaction networks and mechanisms. Appl. Catal. A.

[22] Gunchenko P.A., Li J., Liu B.F., Chen H.Y., Pashenko A.E., Bakhonsky V.V., Zhuk T.S., Fokin A.A. (2018). Aerobic oxidations with N-hydroxyphthalimide in trifluoroacetic acid. Mol. Catal..

[23] Gaster E., Vainer Y., Regev A., Narute S., Sudheendran K., Werbeloff A., Shalit H., Pappo D. (2015). Significant enhancement in the efficiency and selectivity of iron-catalyzed oxidative cross-coupling of phenols by fluoroalcohols. Angew. Chem. Int. Ed..

[24] Shi G., Xu S., Bao Y., Xu J., Liang Y. (2019). Selective aerobic oxidation of toluene to benzaldehyde on immobilized CoOx on SiO_2_ catalyst in the presence of N-hydroxyphthalimide and hexafluoropropan-2-ol. Catal. Commun..

[25] Mostafa M.S., Naga A.O.A.E., Galhoum A.A., Guibal E., Morshedy A.S. (2019). A new route for the synthesis of self-acidified and granulated mesoporous alumina catalyst with superior Lewis acidity and its application in cumene conversion. J. Mater. Sci..

[26] Bao L., Li X.X., Wu Z.W., Yuan X., Luo H.A. (2016). N-hydroxyphthalimide incorporated onto Cu-BTC metal organic frameworks: A novel catalyst for aerobic oxidation of toluene. Res. Chem. Intermed..

[27] Chidambaram M., Sonavane S.U., de la Zerda J., Sasson Y. (2007). Didecyldimethylammonium bromide (DDAB): A universal, robust, and highly potent phase-transfer catalyst for diverse organic transformations. Tetrahedron.

[28] Patil R.D., Fuchs B., Taha N., Sasson Y. (2016). Solvent-free and selective autooxidation of alkylbenzenes catalyzed by Co/NHPI under phase transfer conditions. ChemistrySelect.

[29] Dobras G., Orlińska B. (2018). Aerobic oxidation of alkylaromatic hydrocarbons to hydroperoxides catalysed by N-hydroxyimides in ionic liquids as solvents. Appl. Catal. A.

[30] Melone L., Prosperini S., Ercole G., Pastori N., Punta C. (2014). Is it possible to implement N-hydroxyphthalimide homogeneous catalysis for industrial applications? A case study of cumene aerobic oxidation. J. Chem. Technol. Biotechnol..

