# Supplementary material for: An Investigation of N-Hydroxyphthalimide Catalyzed Aerobic Oxidation of Toluene without Metal Ions in Liquid Phase: Effect of Solvents and Phase Transfer Catalysts
Source: Molecules. 2024 Jun 27;29(13):3066. doi: 10.3390/molecules29133066 (PMC11243731; doi:10.3390/molecules29133066)
Supplement: Supplementary file 1 [file molecules-29-03066-s001.zip › molecules-3071685-supplementary.pdf]

An Investigation of N-Hydroxyphthalimide Catalyzed Aerobic Oxidation of Toluene without Metal Ions in Liquid Phase: Effect of Solvents and Phase Transfer Catalysts

Guojun Shi *, Longsheng Dong and Ya Feng

School of Chemistry and Chemical Engineering, Yangzhou University, Yangzhou 225002, China
* Correspondence: gjshi@yzu.edu.cn; Tel./Fax: +86-514-87937661

1. Effect of solvents on metal-free oxofunctionalization of toluene

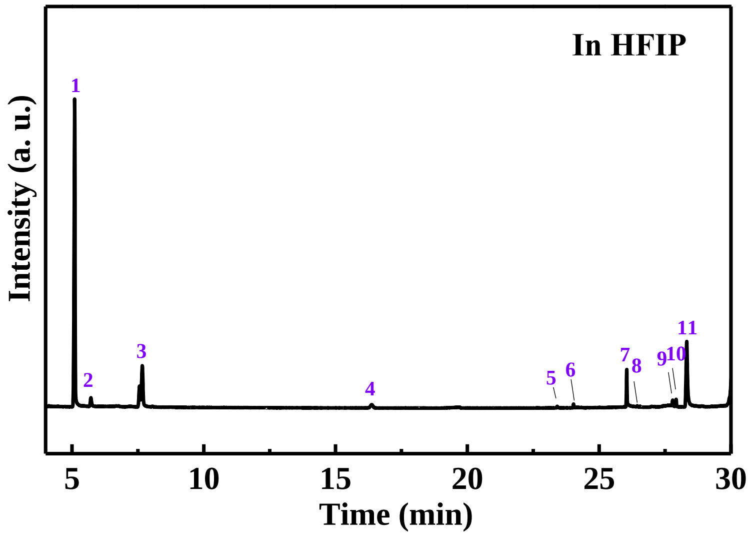

Figure S1 Chromatographic elution curve of the reaction solution in HFIP via GC-MS. Reaction conditions: toluene (2 mmol), HFIP (40 mmol), NHPI (0.2 mmol), O_2 (2 MPa), 90 °C, 5 h. 1: benzaldehyde; 2: phenol; 3: benzyl alcohol; 4: o-benzyl hydroxylamine; 5: phenylmethoxyure; 6: o-phthalic anhydride; 7: phthalimide; 8: 1-benzyl-3-methylbenzene; 9: benzyl benzoate; 10: phenethoxybenzene; 11: N-hydroxyphthalimide.

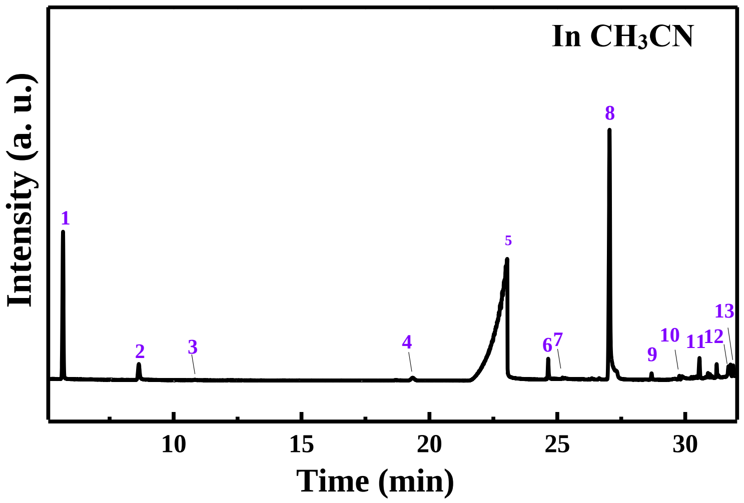

Figure S2 Chromatographic elution curve of the reaction solution of toluene in CH_3CN via GC-MS. Reaction conditions: toluene (2 mmol), CH_3CN (40 mmol), NHPI (0.2 mmol), O_2 (2 MPa), 90 ˚C , 5 h. CH_3CN: acetonitrile; NHPI: N-hydroxyphthalimide; 1: benzaldehyde; 2: benzyl alcohol; 3: benzyl formate; 4: 2-nitrobenzyl alcohol; 5: benzoic acid; 6: o-phthalic anhydride; 7: 2-phenylmethoxycarbonylbenzoic acid; 8: phthalimide; 9: N-hydroxyphthalimide; 10: o-tolyl benzoate; 11: 2-(4-methylphenyl)isoindole-1,3-dione; 12: 2-[(3-methylphenyl)carbamoyl]benzoic acid; 13: N-benzylphthalimide.

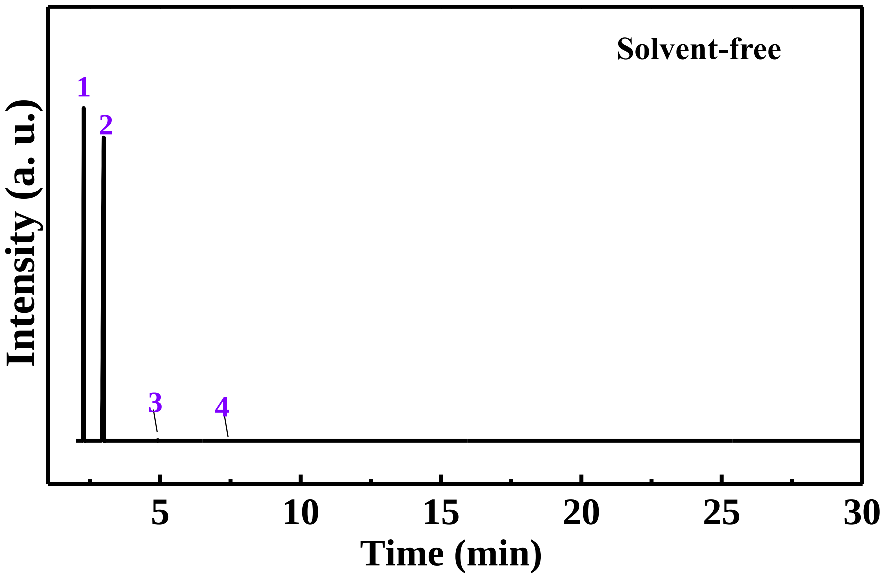

Figure S3 Chromatographic elution curve of the reaction solution of toluene under solvent-free conditions via GC-MS. Reaction conditions: toluene (40 mmol), NHPI (4 mmol), O_2 (2 MPa), 90 ˚C, 5 h. 1: toluene; 2: chlorobenzene; 3: benzaldehyde; 4: benzyl alcohol.

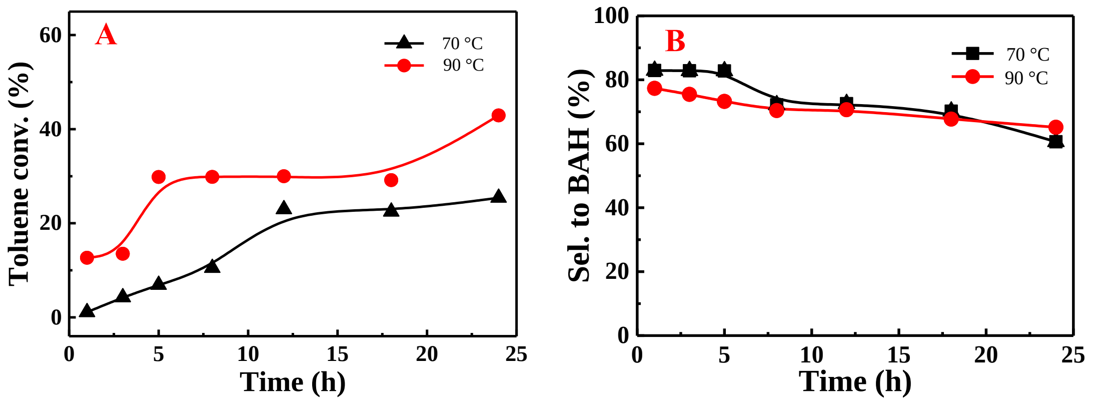

Figure S4 Effect of reaction time on toluene conversion (A) and the selectivity to benzaldehyde (B) in HFIP. Conv.: conversion; Sel.: selectivity; BAH: benzaldehyde.

Table S1. Catalyst controlled experiment in HFIP.

Catalyst	Conversion (%)	Selectivity (%)				
		BAH	BAL	BAC	DBE	Others
-	0.52	100	0.0	0.0	0.0	0.0
NHPI	29.8	73.3	16.9	4.6	5.0	0.2
NHPI, TEMPO[b]	0.0	-	-	-	-	-

[a] Reaction conditions: toluene (2 mmol), HFIP (40 mmol), NHPI (0.2 mmol), O_2 (2 MPa), 90 ˚C, 5 h. [b] TEMPO (2 mmol). NHPI: N-hydroxyphthalimide; HFIP: hexafluoroisopropanol; TEMPO: 2,2,6,6-tetramethylpiperidinooxy; BAH: benzaldehyde; BAL: benzyl alcohol; BAC: benzoic acid; DBE: dibenzyl ether.

2. Role of phase transfer catalysts on metal-free oxofunctionalization of toluene under solvent-free conditions

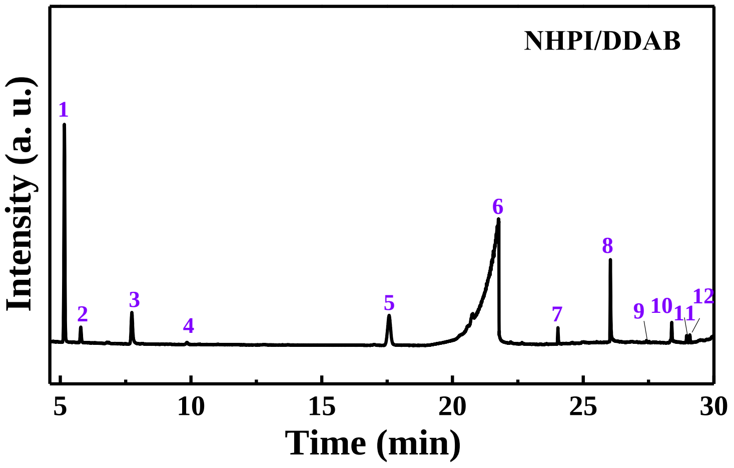

Figure S5. Chromatographic elution curve of the reaction solution of toluene oxidation catalyzed by NHPI/DDAB under solvent-free conditions via GC-MS. Reaction conditions: toluene (40 mmol), NHPI (4 mmol), DDAB (0.2 mmol), O_2 (2 MPa), 90 ˚C, 5 h. NHPI: N-hydroxyphthalimide; DDAB: didecyl dimethyl ammonium bromide; 1: benzaldehyde; 2: benzonitrile; 3: benzyl alcohol; 4: methyl formate; 5: 2-nitrobenzyl alcohol; 6: benzoic acid; 7: o-phthalic anhydride; 8: phthalimide; 9: N-hydroxyphthalimide; 10: (4-methylphenyl)benzoate; 11: benzyl benzoate; 12: o-tolyl benzoate.

Table S2. The conversion and selectivities for selective aerobic oxidation of paraxylene catalyzed by the binary NHPI/DDAB under solvent-free conditions.

Substrate	Conversion (%)	Selectivity (%)			
		p-MBAH	p-MBAL	p-MBAC	Others
Paraxylene	54.2	24.7	10.8	61.5	3.0

Reaction conditions: Reaction conditions: paraxylene (40 mmol), NHPI (4 mmol), DDAB (0.2 mmol), O_2 (2 MPa), 90 ˚C, 5 h. NHPI: N-hydroxyphthalimide; DDAB: didecyl dimethyl ammonium bromide; p-MBAH: p-methyl benzaldehyde; p-MBAL: p-methyl benzyl alcohol; p-MBAC: p-methyl benzoic acid; others: p-Methylbenzyl acetate and p-methylbenzyl formate.
